# Associations between Metformin and Aspirin Use on Cancer Incidence and Mortality in Older Adults.

**DOI:** 10.1093/geroni/igab046.2339

**Published:** 2021-12-17

**Authors:** Suzanne Orchard, Jonathan Broder, Jessica Lockery, Peter Gibbs, Sara Espinoza, Michael Ernst, Robyn Woods, and John McNeil

**Affiliations:** 1 Monash University, Melbourne, Victoria, Australia; 2 The Walter & Eliza Hall Institute of Medical Research, Parkville, Victoria, Australia; 3 University of Texas Health Science Center San Antonio, San Antonio, Texas, United States; 4 University of Iowa, Iowa City, Iowa, United States

## Abstract

Diabetes increases risk of malignancies, and this association increases with age. Metformin may protect against cancer development and progression, but results are mixed and limited to younger cohorts. We examined whether metformin, in the presence or absence of aspirin, reduces incident cancer and cancer-related mortality in older adults. ASPirin in Reducing Events in the Elderly (ASPREE) was a primary prevention trial of daily aspirin vs placebo which enrolled community-dwelling adults from Australia (70+ years) and the US (65+ years for minorities) followed for a median of 4.7 years. Invasive cancer was adjudicated by an expert panel. Cox proportional-hazards models, controlling for age at randomization and known cancer risk factors, were used to analyse the relationship between baseline metformin use, randomized treatment arm, cancer incidence (first in-trial cancer) and mortality. For participants with controlled diabetes, there was a significant reduction in cancer mortality in metformin users compared to nonusers (Adjusted [Adj] HR=0.24, 95%CI=0.07, 0.80), but not for cancer incidence (Adj HR=0.61, 95%CI=0.29, 1.27). For participants with uncontrolled diabetes, there was no significant difference in cancer incidence (Adj HR=0.95, 95%CI=0.66, 1.38) or mortality (Adj HR=1.18, 95%CI=0.62, 2.26) between metformin and non-metformin users. Uncontrolled diabetes, irrespective of metformin use, increased risk of cancer incidence and mortality compared to non-diabetics. Aspirin did not modify the effect of metformin on cancer incidence or mortality. Our findings show that metformin may have protective effects against cancer-related mortality for those older persons whose diabetes is well-controlled, and underscores the importance of diabetes control to minimise cancer risk.

